# Mitochondrial Plasticity and Glucose Metabolic Alterations in Human Cancer under Oxidative Stress—From Viewpoints of Chronic Inflammation and Neutrophil Extracellular Traps (NETs)

**DOI:** 10.3390/ijms25179458

**Published:** 2024-08-30

**Authors:** Hui-Ting Lee, Chen-Sung Lin, Chao-Yu Liu, Po Chen, Chang-Youh Tsai, Yau-Huei Wei

**Affiliations:** 1 Division of Allergy, Immunology & Rheumatology, Department of Internal Medicine, Mackay Memorial Hospital, Taipei 104, Taiwan; htlee1228@gmail.com; 2 Department of Medicine, Mackay Medical College, New Taipei City 252, Taiwan; 3 Division of Thoracic Surgery, Department of Surgery, Taipei Hospital, Ministry of Health and Welfare, New Taipei City 242, Taiwan; doc2765c@ms59.hinet.net; 4 School of Medicine, College of Medicine, National Yang Ming Chiao Tung University, Taipei 112, Taiwan; chaoyuliu6@gmail.com; 5 Center for General Education, Kainan University, Taoyuan City 338, Taiwan; 6 Division of Thoracic Surgery, Department of Surgery, Far Eastern Memorial Hospital, New Taipei City 220, Taiwan; 7 Cancer Free Biotech, Taipei 114, Taiwan; pohan.chen@cancerfree.io; 8 Institute of Clinical Medicine, National Yang Ming Chiao Tung University, Taipei 112, Taiwan; 9 Clinical Trial Center, Division of Immunology & Rheumatology, Fu Jen Catholic University Hospital, New Taipei City 243, Taiwan; 10 Faculty of Medicine, Fu Jen Catholic University, New Taipei City 242, Taiwan; 11 Center for Mitochondrial Medicine and Free Radical Research, Changhua Christian Hospital, Changhua City 500, Taiwan

**Keywords:** Warburg effect, mitochondrial plasticity, oxidative stress, neutrophil, neutrophil extracellular traps (NETs), inflammation

## Abstract

Oxidative stress elicited by reactive oxygen species (ROS) and chronic inflammation are involved both in deterring and the generation/progression of human cancers. Exogenous ROS can injure mitochondria and induce them to generate more endogenous mitochondrial ROS to further perpetuate the deteriorating condition in the affected cells. Dysfunction of these cancer mitochondria may possibly be offset by the Warburg effect, which is characterized by amplified glycolysis and metabolic reprogramming. ROS from neutrophil extracellular traps (NETs) are an essential element for neutrophils to defend against invading pathogens or to kill cancer cells. A chronic inflammation typically includes consecutive NET activation and tissue damage, as well as tissue repair, and together with NETs, ROS would participate in both the destruction and progression of cancers. This review discusses human mitochondrial plasticity and the glucose metabolic reprogramming of cancer cells confronting oxidative stress by the means of chronic inflammation and neutrophil extracellular traps (NETs).

## 1. Redox and Reactive Oxygen Species (ROS)

Synchronized reduction and oxidation (redox), coupled with the gain and loss of electrons, is a common biological reaction in human cells. Proper redox with appropriate electrons transfer can start up a cellular reaction (e.g., glucose metabolism), energy production (e.g., ATP synthesis), or a redox equivalent delivery (e.g., reduced nicotinamide adenine dinucleotide phosphate, NADPH) to keep cells alive or maintain their normal functions. Generally, most human cells tend to situate in a more reduced and less oxidized state [1].

When the cell suffers from a disturbed redox state with inadequate electron transfer, it can result in the formation of unstable reactive oxygen species (ROS), i.e., the incomplete reduction of oxygen. These ROS are usually presented in the forms of superoxide anions (O_2_^•−^), hydrogen peroxide (H_2_O_2_), hydroxyl radicals (^•^OH) or singlet oxygen (^1^O_2_). Although they are unstable and may cause oxidative damage, ROS can be detoxified or eliminated by enzymatic or non-enzymatic pathways in human cells [2]. Thanks to their toxic effect, ROS are utilized by immune cells to destroy invading pathogens [3].

## 2. Glucose Metabolism, Glycolysis, Krebs Cycle, Electron Transport Chain, Oxidative Phosphorylation, Electron Leak and Mitochondrial ROS

**Figure 1:**
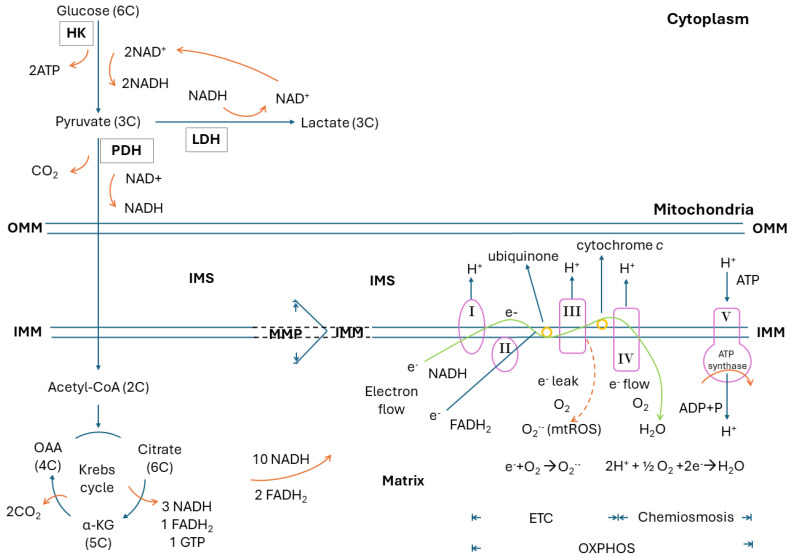
Glucose metabolism through glycolysis, Krebs cycle and OXPHOS to generate ATP, as well as electron leak and mtROS generation, are illustrated. α-KG: alpha-ketoglutarate; FAD: oxidized flavin adenine dinucleotide; FADH_2_: reduced flavin adenine dinucleotide; HK: hexokinase; IMM: inner mitochondrial membrane; IMS: intermembrane space; LDH: lactate dehydrogenase; MMP: mitochondrial membrane potential; mtROS: mitochondrial ROS; NAD^+^: oxidized nicotinamide adenine dinucleotide; NADH: reduced nicotinamide adenine dinucleotide; OAA: oxaloacetate; OMM: outer mitochondrial membrane; OXPHOS: oxidative phosphorylation; PDH: pyruvate dehydrogenase; ROS: reactive oxygen species.

ATP generation, derived through a stepwise oxidation from 6-carbon (6C) glucose (C_6_H_12_O_6_) to 1C CO_2_ and H_2_O, is a typical result of the redox process. Mitochondria are the major intracellular organelles to generate ATP, in which glucose acts as the main source of fuel. In a complete biological oxidation, one molecule of glucose can generate a maximum of 36–38 molecules of ATP (C_6_H_12_O_6_ + 6O_2_ → 6H_2_O + 6 CO_2_ + 36–38 ATP). After uptake into the cell, one molecule of 6C glucose is oxidized to two molecules of 3C pyruvate, which takes place in the cytoplasm through glycolysis by several glycolytic enzymes, with hexokinase (HK) being the first one. In the meanwhile, this glycolytic process can reduce two molecules of oxidized nicotinamide adenine dinucleotide (NAD^+^) into reduced nicotinamide adenine dinucleotide (NADH) and generate two molecules of ATP. In subsequent oxidation by pyruvate dehydrogenase (PDH), one molecule of 3C pyruvate is oxidized to one molecule of 2C acetyl-CoA, which can couple with the reduction of one molecule of NAD^+^ to NADH and a release of one molecule of CO_2_ to join the Krebs cycle through a 4C oxaloacetate (OAA). At the other end of this cycle, a 6C citrate would eventually arise in the mitochondria. In detail, the 6C citrate would be oxidized to 5C alpha-ketoglutarate (α-KG) and then 4C OAA consequently, with a generation of three molecules of NADH and one molecule of reduced flavin adenine dinucleotide (FADH_2_), as well as a release of two molecules of CO_2_. Finally, ATPs are generated by means of oxidative phosphorylation (OXPHOS), which includes electron transport accomplished by the electron transport chain (ETC) with the pump of proton (H^+^) into intermembrane space (IMS) through mitochondrial respiratory complex I, III, and IV, as well as chemiosmosis. In the former, oxygen is the final electron receptor, which together with a proton would result in the generation of one molecule of H_2_O (2H^+^ + 2e^−^ + $\frac{1}{2}$ O_2_ → H_2_O, a highly oxygen-dependent process). In the latter, chemiosmosis is elicited by proton gradient to synthesize ATP through ATP synthase. All the 10 molecules of NADH and 2 molecules of FADH_2_ generated during glycolysis and the Krebs cycle derived from 1 molecule of glucose can be re-oxidized to NAD^+^ and FAD, respectively, and result in the generation of 34 molecules of ATP (1 molecule of NADH and 1 molecule of FADH_2_ would create 3 and 2 molecules of ATP, respectively). Along with the 2 molecules of ATP from glycolysis and 1 molecule of GTP (=ATP) from each Krebs cycle, a maximum of 36–38 molecules of ATP would be generated from 1 molecule of glucose. The NAD^+^ and FAD are then re-utilized in other dehydrogenases of glucose metabolism [4,5]. Figure 1 shows the whole process of these biochemical reactions.

The ETC, which is located in the inner mitochondrial membrane (IMM), consists of mitochondrial respiratory enzyme complexes I, II, III and IV, along with the electron transporters ubiquinone and cytochrome *c*. There are two ways to execute electron transport in the ETC. The former is complex I/III/IV that oxidizes NADH to NAD^+^, resulting in the reduction of O_2_ to H_2_O with a yield of three ATPs. The latter is complex II/III/IV that oxidizes FADH_2_ to FAD with a reduction of O_2_ to H_2_O and a yield of two ATPs. During an electron flow transferred through respiratory complexes in the inner mitochondrial membrane (IMM), complexes I, III and IV drive protons to the IMS, an area between the outer and inner mitochondrial membranes, to create a proton gradient for chemiosmosis. Thus, a mitochondrial membrane potential (MMP) representing the magnitude of mitochondrial membrane polarization is established across the IMM [6]. Physiologically, approximately 0.2–2.0% of these electrons leak out and interact with oxygen directly to produce O_2_^•−^ (e^−^ + 1/2 O_2_ → O_2_^•−^) [7]. Therefore, mitochondria are a major source of endogenous ROS, the mitochondrial ROS (mtROS), in human cells. These mtROS participate in human aging and aging-related diseases [8]. The amount of mtROS is highly related to the degree of MMP and the extent of polarization of IMM [9].

When the cells suffer from oxygen shortage or mitochondrial respiratory dysfunction, pyruvate can be reduced to lactate by lactate dehydrogenase (LDH) in the cytoplasm without entering the mitochondria. This reduction is accompanied by the oxidation of NADH to NAD^+^, which can complete the glycolysis circuit (Glucose + 2 ADP^3−^ + 2 NADH → 2 Lactate + 2 ATP^4−^ + 2 NAD^+^). Although lactate fermentation generates only two molecules of ATP per molecule of glucose, it provides a faster method of energy supply than does mitochondrial respiration [4,5].

Nevertheless, there are some exceptions in human blood cells, e.g., neutrophils, peripheral blood monocytes, erythrocytes or platelets. These cells mainly rely on glycolysis to generate ATPs, no matter whether they have or do not have mitochondria [10,11]. Noteworthily, their mitochondria mainly deal with the production of endogenous mtROS to trigger immune response, as has been uniquely shown in neutrophils [12]. Reithofer et al. [13] demonstrated that mitochondria with a higher MMP and hyper-polarization generate more mtROS that involve neutrophil activation.

## 3. Chronic Inflammation and Human Cancer

**Figure 2:**
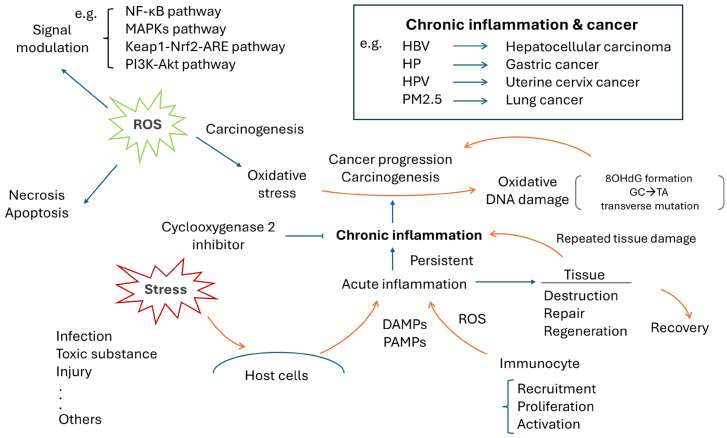
Schematic illustration shows the underlying mechanism of chronic inflammation in carcinogenesis and cancer progression. 8-OHdG: 8-hydroxy-2′-deoxyguanosine; A: adenine; C: cytosine; DAMPs: damage-associated molecular patterns; G: guanine; HBV: hepatitis B virus; HP: Helicobacter pylori; HPV: human papillomavirus; Keap1-Nrf2-ARE: Kelch-like ECH-associated protein 1 (Keap1)/nuclear factor erythroid 2-related factor 2 (Nrf2)/antioxidant response elements (ARE); MAPK(s): mitogen-activated protein kinase(s); NF-κB: nuclear factor kappa-light-chain-enhancer of activated B cells; PAMPs: pathogen-associated molecular patterns; PI3K-Akt: phosphoinositide-3-kinase (PI3K)-Akt; PM2.5: particulate matter ≤ 2.5 µm; ROS: reactive oxygen species; T: thymine.

As demonstrated in Figure 2, when tissues suffer from damage caused by infection, toxic substances, mechanical injury or other stressors, an inflammatory process is triggered in response to the chemotactic substances such as damage-associated molecular patterns (DAMPs) or pathogen-associated molecular patterns (PAMPs), that are released by damaged cells or invading organisms. This process is characterized by the recruitment, proliferation and activation of a variety of immune cells, including neutrophils, macrophages, NK cells, B cells, T cells and fibroblasts, as well as endothelial cells, all of which might also be involved in tissue repair and regeneration. An inflammation is a normal physiological response to protect and repair damaged tissues, but it should not persist longer than required. Otherwise, harmful consequences can ensue [14].

A persistent inflammation can cause repeated tissue damage, tissue repair and tissue regeneration, which evolve into a slowly “burning status” and become a chronic inflammation. Previous studies demonstrated that chronic inflammation can cause oxidative DNA damage to form 8-hydroxy-2′-deoxyguanosine (8-OHdG), which might become carcinogenetic [15]. This scenario is supported by the clinical evidence of the relation between *Helicobacter pylori* (HP) and gastric cancer [16], *Clonorchis sinensis* and cholangiocarcinoma [17], human papillomavirus (HPV) and cervical cancer [18], hepatitis B virus (HBV) and hepatocellular carcinoma (HCC) [19], sun exposure and skin cancer [20] or particulate matters ≤ 2.5 µm (PM2.5) in air pollutants and lung cancer [21]. On the other hand, the anticancer effect elicited by an anti-inflammation therapy, the cyclooxygenase 2 inhibitor, has further supported this viewpoint [22]. An imbalance or disturbance between pro- and anti-inflammation might play an important role in cancer formation and progression. Thus, ROS-associated oxidative damages is speculated to be a major contributor that links chronic inflammation and carcinogenesis [23].

## 4. ROS, Oxidative Stress and Cancer

Oxidative stress indicates an increase in ROS production or a decrease in ROS elimination. ROS overproduction may be caused by exogenous stimuli (e.g., infection or inflammation), endogenous metabolic alterations (e.g., diabetes mellitus [DM] or metabolic syndrome) or other factors to disrupt redox homeostasis. Different ROS levels might cause various forms of cell responses. An abrupt and sudden increase in ROS to an extreme extent could cause cell death by means of necrosis, apoptosis or other factors. Moderate or sub-lethal ROS levels can act as mediators to trigger several signal pathways for maintaining or promoting cell functions and then resuming the steady redox status. Nevertheless, a persistent high ROS level driven by repetitive stimuli might cause carcinogenesis or cancer progression [24].

ROS acting as a signal modulator has been reported in several signaling pathways, including the nuclear factor kappa-light-chain-enhancer of activated B cells (NF-κB), mitogen-activated protein kinases (MAPKs), Kelch-like ECH-associated protein 1 (Keap1)/nuclear factor erythroid 2-related factor 2 (Nrf2)/antioxidant response elements (ARE)(Keap1-Nrf2-ARE), and phosphoinositide-3-kinase (PI3K)-Akt (PI3K-Akt) pathways. NF-κB is a transcription factor that modulates gene expression in a variety of cellular processes, e.g., innate immune response, embryogenesis and organ development, cell proliferation and apoptosis. Disturbed NF-κB might be associated with cancer or human diseases such as arthritis, inflammation or degenerative diseases. ROS can both activate and inhibit the NF-κB pathway through (1) targeting the upstream kinases, the IkappaB (I-κB) kinase (IKK), NF-κB-inducing kinase (NIK) and Akt and (2) facilitating or inhibiting the degradation of I-κB or the modulation of the nuclear translocation and DNA binding of the transcription factor by modifying the NF-κB heterodimers [25]. MAPK is involved in a series of transductions of biological signals from the cell membrane to the nucleus. There are several well-identified subgroups of MAPKs in mammalian cells, e.g., the extracellular signal regulated kinases (ERKs), the c-Jun N-terminal kinases (JNKs) and p38 MAPK. They belong to serine/threonine kinases that are activated through a cascade of phosphorylation events. Interestingly, it was reported that ROS can interfere with ERKs [26], JNKs [27] and p38 [28] to cause human diseases. The Nrf2-Keap1 system is an evolutionarily conserved intracellular defense mechanism to counteract oxidative stress. Dysregulation can lead to several inflammatory diseases including cancer, Alzheimer’s disease (AD), Parkinson’s disease (PD), and DM. A recent study showed the Nrf2-Keap1-ARE pathway is influenced by different levels of ROS [29]. ROS causes Nrf2 to dissociate from Keap1 and translocate to the nucleus to bind ARE, to induce the expression of antioxidant and metabolic genes [29]. The PI3K-Akt pathway participates in various types of cancer. It controls cancer cell survival, metastasis and metabolism [30]. ROS can activate PI3K-Akt and promote its downstream pro-tumorigenic signaling cascades [31]. In brief, ROS can regulate several signaling pathways (Figure 2, left upper panel).

Without efficient detoxification, ROS can cause oxidative DNA damage. The formation of 8-OHdG, oxidative damage to guanine (G), is the most common one and is highly mutagenic. If it is not repaired and accumulates in damaged tissues, 8-OHdG will pair erroneously with adenine (A) instead of cytosine (C). This may cause a G:C to thymine (T):A transversion mutation and initiate subsequent carcinogenesis [32], as shown in Figure 2. A meta-analysis has demonstrated that a high 8-OHdG accumulation is associated with a poor prognosis in solid cancers, including hepatocellular carcinoma, ovarian cancer, esophageal cancer, lung cancer, bladder cancer and renal cancer, as well as colorectal cancer [33]. The associations of the exposure to tobacco smoke and oxidative damage (8-OHdG) in human lung cancers have been reported [34,35]. Recently, more attention has been paid to the links between PM2.5, oxidative damage and lung cancer [21].

## 5. NETs, ROS and Host Oxidative Damages

**Figure 3:**
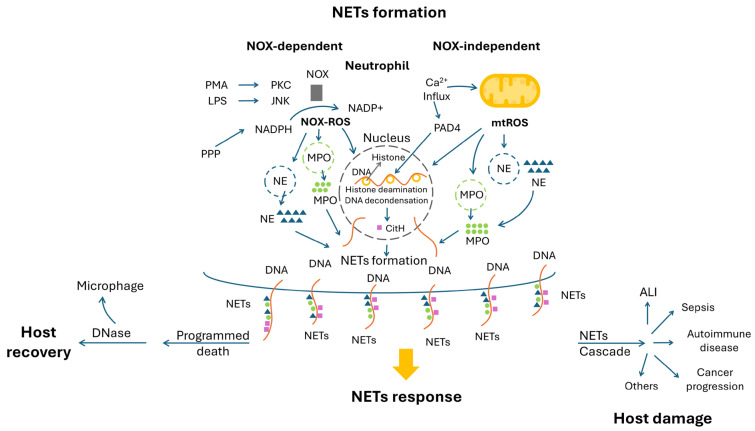
NETs formation includes NOX-dependent (through NOX-ROS) and NOX-independent (through mtROS) pathways. Most NETs are eliminated uneventfully after completing their tasks (left lower part). A NETs cascade might happen and cause several adverse effects to the host, including cancer progression (right lower part). ALI: acute lung injury; CitH: citrullinated histone; JNK: c-Jun N-terminal kinase; LPS: lipopolysaccharide; MPO: myeloperoxidase; mtROS: mitochondrial ROS; NE: neutrophil elastase; NETs: neutrophil extracellular traps; NOX: NADPH oxidase; PKC: protein kinase C; PMA: Phorbol 12-myristate 13-acetate; PPP: Pentose phosphate pathway; PAD4: protein arginine deiminase 4; ROS: reactive oxygen species.

Neutrophils, with a key role in human innate immunity, can respond to and destroy invading pathogens such as bacteria, fungi and viruses or chemical substances, e.g., gouty crystal or air pollutants, by means of phagocytosis, degranulation or neutrophil extracellular traps (NETs) [36]. Among them, NETs formation requires the generation of endogenous ROS from neutrophils per se, which can be classified into (a) NADPH oxidase (NOX)-dependent and (b) NOX-independent types [36]. During NETs formation, stimulated neutrophils extrude their DNA, histones and granule enzymes, e.g., myeloperoxidase (MPO) and neutrophil elastase (NE), to the outer surface of their cell membrane to entangle pathogens or chemical substances. MPO can generate more ROS to exert a higher toxicity [37]. NE is a major protease involved in microbicidal activity and an important factor in promoting inflammation [38]. Quite similar but not the same as apoptosis or necrosis, these neutrophils “aroused” with NETs formation can eventually result in programmed cell death after completing their task, a process that may be called “netosis” [39]. Figure 3 shows the mechanisms underlying these intricate reactions.

### 5.1. NOX-Dependent NET Formation

This type of NET formation mainly depends on ROS produced by NOX. After assembling, NOX enzymes oxidize NADPH to generate ROS, which can disintegrate the membrane of cytoplasmic granules, mainly NE and MPO, and the membrane of the nucleus. The free NE and MPO thus enter the nucleus and cleave the histone to de-condensate chromatin. Decorated with NE, MPO or other granular enzymes, the de-condensated DNA can be released to the extracellular environment as NETs to execute their anti-microbial function [36].

The assembly of NOX requires its phosphorylation to be executed by upstream protein kinase C (PKC) or JNK, which are two different pathways elicited by different stimulants. Following the entry of a non-physiological agonist Phorbol 12-myristate 13-acetate (PMA), a diacylglycerol mimetic, the Ca^2+^ ions released from the endoplasmic reticulum enter the cytosol. Then, PKC can be activated to phosphorylate gp91phox/NOX2, and the function of NOX is gained [40]. Following the binding of lipopolysaccharides (LPS) from Gram-negative bacterial infection to toll-like receptor 4, the JNK can phosphorylate gp91phox/NOX2, and induce NOX-dependent NET formation [41].

### 5.2. NOX-Independent NET Formation

NOX-independent NET formation requires the influx of Ca^2+^ ions, the generation of mtROS and the deamination executed by protein arginine deiminase 4 (PAD4). Through Ca^2+^ ionophores, such as ionomycin, the Ca^2+^ influx can be initiated. PAD4, which is present in quantity in the cytosol, can bind with Ca^2+^ and translocate into the nucleus of neutrophils to deaminate the histone arginine residues harboring a positive charge into neutral citrulline as citrullinated histone (CitH). This charge eversion eventually results in the de-condensation of chromatin.

Similar to apoptosis that is induced by the Ca^2+^ influx to mitochondria to generate mtROS [42], the mtROS inside neutrophils can disrupt cellular membranous structures. mtROS can be produced through activation of the SK channel (Ca^2+^-activated K^+^ channel of small conductance) to induce a Ca^2+^ influx to result in NET formation [43].

### 5.3. Maladaptive Roles of NET-Related Tissue Damage and Cancer Biology

After completing their tasks, the circulated or in situ NET remnants would be cleaved by deoxyribonucleases (DNases), including DNase I and DNase II, through the hydrolysis of DNA and eventually be degraded by macrophages [44]. Without adequate clearance, NETs, as well as MPO and NE, might arouse another sequence of oxidative damage to the host tissues. Associated host cell damage has been reported in sepsis [45], acute lung injury (ALI), autoimmune diseases, e.g., systemic lupus erythematosus [46], rheumatoid arthritis [47], type 1 diabetes mellitus [48], gouty arthritis [49], inflammatory bowel disease [50] and metabolic diseases including type 2 diabetes mellitus [51] and obesity [52]. Moreover, NETs have been implicated in cancer progression, metastasis and therapy resistance [53]. As a double-edged sword to human cancers, whether NETs are a foe, causing oxidative damage to trigger cancer cell apoptosis, or a friend, acting as a modulator to promote cancer growth, has remained obscured [54].

## 6. NETs, Oxidative Toxicity, Mitochondrial Dysfunction and Warburg Effect in Human Cancers

**Figure 4:**
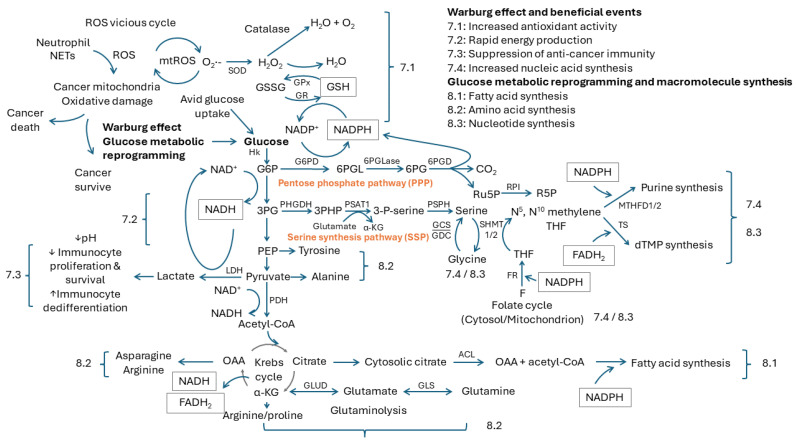
Illustration of NETs, ROS, cancer mitochondrial oxidative damage and altered glucose metabolism, including the Warburg effect and glucose metabolic reprogramming, in human cancers is shown. Beneficial events from the Warburg effect are sketched and described in text sections, including 7.1: Increased antioxidant activity, 7.2: Rapid energy production, 7.3: Suppression of anticancer immunity and 7.4: Increased nucleic acid synthesis. Synthesis of macromolecules through glucose metabolic reprogramming is illustrated and described in text sections, including 8.1: Fatty acid synthesis, 8.2: Amino acid synthesis and 8.3: Nucleotide synthesis. 3PG: 3-phosphoglycerate; 3PHP: 3-phosphate hydroxypyruvate; 3-P-serine: 3-phosphoserine; 6PGD: 6-phosphogluconate dehydrogenase; 6PGL: 6-phosphogluconolactone; ACL: ATP-citrate lyase; α-KG: alpha-ketoglutarate; F: Folate; FADH_2_: reduced flavin adenine dinucleotide; FR: folate reductase; G6P: glucose-6-phosphate; G6PD: glucose 6-phosphate dehydrogenase; GCS: Glycine cleavage system; GDS: glycine decarboxylase complex; GLS: glutaminase; GLUD: glutamate dehydrogenase; GPx: glutathione peroxidase; GR: glutathione reductase; GSH: reduced glutathione; GSSG: oxidized glutathione; HK: hexokinase; LDH: lactate dehydrogenase; MTHFD: methylenetetrahydrofolate dehydrogenase/cyclohydrolase; mtROS: mitochondrial ROS; NAD^+^: oxidized nicotinamide adenine dinucleotide; NADH: reduced nicotinamide adenine dinucleotide; NADP^+^: oxidized nicotinamide adenine dinucleotide phosphate; NADPH: reduced nicotinamide adenine dinucleotide phosphate; NETs: neutrophil extracellular traps; OAA: oxaloacetate; PDH: pyruvate dehydrogenase; PEP: phosphoenolpyruvate; PHGDH: phosphoglycerate dehydrogenase; PPP: pentose phosphate pathway; PSAT1: phosphoserine aminotransferase; PSPH: 1–3-phosphoserine phosphatase; R5P: ribose 5-phosphate; ROS: reactive oxygen species; RPI: 5-phosphate isomerase; Ru5P: ribulose 5-phosphate; SHMT: serine hydroxymethyl transferase; SOD: superoxide dismutase; SSP: serine synthesis pathway; THF: tetrahydrofolate; TS: thymidylate synthase.

Neutrophil-NETs can elicit toxic ROS to destroy cancer cells to achieve anticancer effects [55,56]. Lethal ROS generated from neutrophil-NETs can disrupt cancer mitochondria to induce their apoptotic or necrotic death [57,58]. The dysfunction of cancer mitochondria was first described by Dr. Warburg, who proposed that mitochondrial dysfunction might be at the origin of cancer. He found that cultured cancer tissues exhibited an avid glucose uptake and a profound lactate production even in the situation of sufficient oxygen supply. In the meanwhile, the proportion of ATP generated by glycolysis was increased, but that from respiration was decreased. From the viewpoint of energy supply, the amplified glycolysis from glucose to lactate is supposed to compensate for the defective mitochondria in cancers [59,60]. This observation is called the Warburg effect, and the phenomenon of amplified glycolysis has been applied in positron emission tomography (PET) scans to detect human cancers [61]. During the past decades, the dysfunction of cancer mitochondria, as well as amplified glycolysis, have been appraised in several human cancers [62,63,64]. Inside the tumors, there seems to be a tug of war between mitochondrial damage to trigger death and amplified glycolysis to keep cells alive (Figure 4, left upper part).

## 7. Beneficial Events from the Warburg Effect

### 7.1. Increased Antioxidant Activity

Mitochondrial damage can cause a vicious circle between ROS production, oxidative damage and tumor progression in human cancers [65]. There are several enzymes and non-enzyme molecules participating in the detoxification of ROS. The moment superoxide anions (O_2_^•−^) are formed, the superoxide dismutase (SOD), including MnSOD in the mitochondria or Cu/ZnSOD in the cytoplasm, can reduce them to H_2_O_2_. H_2_O_2_ is further metabolized to H_2_O and O_2_ by catalase, or to H_2_O by glutathione peroxidase (GPx) with the help of reduced glutathione (GSH). Glutathione is composed of glycine, glutamine and cysteine. Specifically, GPx can oxidize GSH (glutathione in reduced form) to GSSG (glutathione in oxidation form) to offer the reducing equivalents to reduce H_2_O_2_ to H_2_O (Figure 4, 7.1). A sufficient pool of GSH plays a pivotal role in maintaining the antioxidant capacity of human cells [66].

GPx acts in the downstream of the enzymatic antioxidant pathway; therefore, the clinical relevance of GPx expression and the GSH pool or GSH/GSSG ratio in human cancers have been extensively explored. A high GPx-4 or GPx-1 expression has been reported as a poor prognostic variable in lung cancer and colon cancer, respectively [67,68]. Usually, the GSH/GSSG ratio is used as an indicator to reflect oxidative stress, and it has been applied to compare the oxidative stress level among patients with different types of pediatric tumors [69] or as a tumor marker of colorectal cancer [70]. Compared to healthy controls, patients with head and neck cancers had a lower plasma GSH/GSSG ratio, suggesting a higher oxidative stress due to an increased consumption of GSH [71].

The replenishment of GSH from GSSG requires a supply from NADPH, which is generated from the pentose phosphate pathway (PPP) coupled with glycolysis. PPP consists of an irreversible oxidative phase and reversible non-oxidative phase, and it bifurcates from the glycolytic process through glucose-6-phosphate (G6P), which is the first intermediate after glycolysis. In the oxidative phase of PPP, glucose-6-phosphate dehydrogenase (G6PD) oxidizes G6P to 6-phosphogluconolactone (6PGL), which can synchronize the reduction from oxidized nicotinamide adenine dinucleotide phosphate (NADP^+^) to NADPH [72]. Under hydrolysis by 6-phosphogluconolactonase (6PGLase), the 6PGL is converted to 6-phosphogluconate (6PG). Finally, 6-phosphogluconate dehydrogenase (6PGD) oxidizes 6PG to produce ribulose 5-phosphate (Ru5P), NAPDH and CO_2_. Overall, PPP contributes the largest cytosolic pool of NADPH to offer sufficient reducing equivalents in mammalian cells. With the assistance of glutathione reductase (GR), the GSSG can be reduced to GSH, coupled with the oxidation from NADPH to NADP^+^ to replenish the GSH pool [72]. This biochemical flow is illustrated in Figure 4, 7.1.

G6PD deficiency is an X-linked recessive disorder, which is characterized by hemolysis after an infection or upon consumption of specific medications, and it is speculated that affected people may have a suboptimal antioxidant capacity [73]. As a result, some researchers demonstrated a lowered risk of cancer progression in patients with a G6PD deficiency attributable to a decreased anti-ROS activity in the cancer tissues of these patients [74]. In cancer biology, a higher G6PD expression can cause a higher antioxidant activity in cancer cells and correlate to a poor prognosis in several human cancers [75]. It was reported that the depletion of NADPH might result in cancer growth inhibition in prostate cancer [76]. Similarly, the depletion of NADPH in triple-negative breast cancer can enhance the anti-PDL1 effect [77]. In aneuploid cancer cells, a high level of NADPH can promote chromosome segregation and tumor progression [78]. It is conjectured that an amplified glycolysis could contribute to an increased antioxidant capacity through NADPH replenishment and prevent ROS damage in human cancers.

This phenomenon could also account for the role of glycolysis in neutrophils. In addition to avoiding the ROS-related oxidative damage to neutrophils, NADPH from PPP further serves as the substrate of NOX to generate the ROS required for NOX-dependent NET formation [36].

### 7.2. Rapid Energy Production

From the viewpoint of energy expenditure, aerobic glycolysis could offer a faster ATP supply than mitochondrial respiration as described in the above paragraph (Figure 1). However, the cell would be situated in a less reduced or a more oxidized status. The cytoplasmic redox between NAD^+^ and NADH plays a key role. During the oxidation from glucose to pyruvate, it will couple the reduction from two molecules of NAD^+^ to two molecules of NADH with producing a net of two molecules of ATP. For cells harboring intact mitochondria, the NADH and pyruvate can undergo a series of redox reactions and OXPHOS to generate sufficient ATP and H_2_O. H_2_O is much more reduced than NADH, and the cells would be in a lower oxidative stress status. For cancer cells with impaired mitochondrial function, the further reduction of pyruvate to lactate is required, because this will synchronize the oxidation from NADH to NAD^+^ (Figure 1 and Figure 4, 7.2). NAD^+^ is critical to maintaining the glycolytic circuit and its ATP production [79].

Similar to GSH and NADPH, NADH can be kept at a reasonable higher level in some cancers [80]. Studies have demonstrated that an increased pool of NADH plus NAD^+^ might promote colon cancer progression [81], or a balanced NAD^+^ to NADH ratio might inhibit breast cancer progression [82]. Besides rapid ATP production, an amplified glycolysis to lactate formation would maintain the favorable redox status of cancer cells.

### 7.3. Suppression of Anticancer Immunity

Usually, lactate cannot be utilized by cancer cells or their surrounding tissues and it would be dumped into the bloodstream or surrounding third space as waste. This would cause lactate acidosis of the local environment around the cancers, which is a key mechanism for neoplastic cells to escape from the attack of host immune cells (Figure 4, 7.3).

First, the local lactic acid environment would inhibit the proliferation and survival of immune cells. During active immune status, immune cells mainly rely on aerobic glycolysis, like the Warburg effect, to maintain the proliferation and expansion of immune cells [10]. These immune cells need to export the lactate wastes to the extracellular environment through specific transporters (e.g., monocarboxylate transporter 1, MCT1) [83]. However, the efficiency of exporting lactic acid depends on the concentration gradient of extracellular lactate. High levels of environmental lactic acid can block the transporter activity, thus preventing the discharge of lactic acid and inhibiting immunocyte proliferation. Moreover, a study showed that concentrations of lactate above 20 mM can reduce the number and activity of immunocytes by causing apoptosis both in vitro and in vivo [84], thus leading to the immune evasion of cancer cells.

Second, accumulated lactic acid can induce the de-differentiation of immunocytes through histone lactylation [85]. De-differentiation is a transient process by which cells become less specialized and return to an earlier cell state within the same lineage [86]. A recent study demonstrated that lactic acid can directly bind to histone lysine in lactylation (Kla) sites to serve as an epigenetic regulator and then to stimulate the expression of specific genes in immune cells [85]. Then, multiple cell types are de-differentiated to less specialized immune cells. It could explain why so many immune cells lose their anticancer abilities in the high-lactate microenvironment. Some studies have demonstrated that lactate can induce the stemness property in cancer cells, which causes the stronger propagation of human cancers [87,88].

LDH is the main enzyme that converts pyruvate to lactate, and its high expression seems an unfavorable predictor. As shown in the Human Protein Atlas (https://www.proteinatlas.org/ENSG00000134333-LDHA/pathology, accessed on 20 August 2024) and the literature [89], high LDHA expression correlates with a poor prognosis in several human cancers. A recent study also demonstrated that LDHA can preserve the stemness properties of breast cancer [90].

### 7.4. Increased Nucleic Acid Synthesis

Cancer growth requires ample nucleic acids. Nucleotide, the unit of nucleic acid, is made from ribose 5-phosphate (R5P); nucleobase, including A, T, uracil (U), G or C; and phosphate. To synthesize nucleotides, PPP and serine synthesis pathway (SSP) from the bifurcations of glycolysis through G6P and 3-phosphoglycerate (3PG) are quite important. These are described in the following and illustrated in Figure 4, 7.4.

In human cells, the Ru5P generated from the oxidative phase of PPP would be reversibly converted to R5P and xylulose 5-phosphate (Xu5P) by ribose 5-phosphate isomerase (RPI) and ribulose 5-phosphate epimerase (RPE), respectively. R5P is a building block for nucleic acid synthesis, and is essential for the survival and proliferation of cancer cells [91].

The synthesis of nucleobase, including pyrimidine or purine, requires cooperation from the folate (F) cycle. Through folate reductase (FR) and NADPH, which is from PPP, to act as an electron donor, the folate would be reduced to dihydrofolate (DHF) and further tetrahydrofolate (THF) in steps. Using serine, which is from SSP [92], to act as the donors for the hydroxyl and methyl groups transferred by serine hydroxymethyl transferase (SHMT), the THF would be converted to N^5^, N^10^ methylene THF, whereas serine would be degraded to glycine simultaneously. N^5^, N^10^ methylene THF is the hub to either pyrimidine or purine biosynthesis [93].

With the consumption of NADPH derived from PPP, which acts as electron donor, and water molecules in order, the N^5^, N^10^ methylene THF would be reduced to N^5^, N^10^ methenyl THF and hydrolyzed to N^10^ formyl THF stepwise by methylenetetrahydrofolate dehydrogenase/cyclohydrolase (MTHFD). N^10^ formyl THF would participate in purine synthesis. Using FADH_2_ from the Krebs cycle to serve as the electron donor, and the involvement of N^5^, N^10^ methylene THF, the dTMP would be synthesized from dUMP by thymidylate synthase (TS) [93].

In glycolysis, 3PG is an intermediate product after G6P and is a precursor for SSP. First, 3PG is oxidized to 3-phosphate hydroxypyruvate (3PHP) by phosphoglycerate dehydrogenase (PHGDH), which is the rate-limiting enzyme of SSP. Second, after the joining of glutamate, 3PHP is catalyzed to 3-phosphoserine (3-P-serine) and α-KG by phosphoserine aminotransferase (PSAT1). The 3-P-serine is dephosphorylated to serine by 1–3-phosphoserine phosphatase (PSPH) [92]. As described above, the serine would participate in the synthesis of nucleobase.

Among PPP, SSP and the folate cycle in nucleotide biosynthesis, several key enzymes, including RPI, PHGDH and SHMT, have been evaluated in human cancers. RPI isomerase A (RPIA) can promote the formation of colorectal cancers [94], and was demonstrated as a poor prognostic variable in human liver and renal cancer (Human Protein Atlas, https://www.proteinatlas.org/ENSG00000153574-RPIA/pathology, accessed on 20 August 2024). A higher expression level of PHGDH was associated with a shorter survival in endometrial cancer [95], breast cancer [96] and lung cancer [97], respectively. A higher SHMT2 expression can predict unfavorable outcomes in multiple cancers, including liver cancer, esophageal cancer, gastric cancer, pancreatic cancer and breast cancer, as demonstrated in a systematic review and meta-analysis [98].

## 8. Glucose Metabolic Reprogramming and Macromolecule Synthesis

Besides the production of ATP to keep cells alive and the generation of mtROS to induce apoptotic cell death, mitochondria act as a hub for the synthesis of macromolecules, including fatty acids and amino acids, as well as nucleic acids, from the amplified glycolysis of the Warburg effect to the off-shunts of the Krebs cycle. This is referred to as glucose metabolic reprogramming in cancer mitochondria [99,100]. It is described in the following and illustrated in Figure 4, 8.1–8.3.

### 8.1. Fatty Acid Synthesis

Acetyl-CoA, derived from pyruvate during glycolysis, is necessary for and involved in the first step of fatty acid synthesis in the cytosol. The majority of acetyl-CoA is produced inside the mitochondria from pyruvate in human cells, but it cannot be exported to the cytosol directly. To synthesize fatty acids, it is important to convert the mitochondrial acetyl-CoA to cytosolic acetyl-CoA. After entering the Krebs cycle to join with OAA, the downstream citrate can be transported from mitochondrion out to the cytosol and then it can be broken into acetyl-CoA and OAA by ATP-citrate lyase (ACL). Therefore, it can participate in fatty acid synthesis. A high glucose concentration would hasten the production of acetyl-CoA. During the process of fatty acid synthesis, it also requires the reducing equivalents of NADPH supplied from PPP. In other words, an increase in glucose uptake and glycolysis is highly related to fatty acid synthesis [101].

High ACL expression has been associated with an unfavorable outcome in gastric cancer [102], epithelial ovarian cancer [103], acute myeloid leukemia [104] and lung cancer [105]. Therefore, some ACL inhibitors have been developed to treat human cancers [106].

### 8.2. Amino Acid Synthesis

Non-essential amino acids (NEAAs) can be synthesized through glycolysis, including 3PG for serine, phosphoenolpyruvate (PEP) for tyrosine and pyruvate for alanine, and from intermediates of the TCA cycle including OAA for asparagine and arginine and α-KG for arginine, proline, glutamate and glutamine [107]. Mitochondria play an important role in the synthesis of amino acid for the rapid growth of cancer cells [108].

In the Krebs cycle, the conversion of α-KG from isocitrate catalyzed by isocitrate dehydrogenase (IDH) is an irreversible step. Alpha-KG plays a pivotal role in the homeostasis of the amino acid pool in human cells [109]. To sustain the Krebs cycle, a sufficient supply of α-KG is important and critical, and it could come from either intra-cycle isocitrate (an irreversible process) or extra-cycle glutamate (a reversible process). Through hydrolysis by glutaminase (GLS), glutamine can be converted to glutamate. Followed by deamination by glutamate dehydrogenase (GLUD) or the SSP as described above, the glutamate could join the Krebs cycle in the form of α-KG. Alpha -KG can enter the TCA cycle to generate NADH, to provide reducing equivalents for the ETC and subsequent OXPHOS. This process is called glutaminolysis, and it can supply energy and synthesize amino acid in human cancer cells [109,110].

A high GLS expression was reported to be a poor prognostic factor in node-positive triple-negative breast cancer patients with a high level of tumor-infiltrating lymphocytes [111] and in liver cancer [112]. GLS expression is required for tumor growth, invasion and metastatic colonization in colorectal cancer [113]. The GLUD was elevated in low-grade Luminal/ER^+^ breast cancer [114], and a high GLUD expression has been reported as a poor prognostic variable in colorectal cancer [115].

### 8.3. Nucleotide Synthesis

Because mitochondria harbor their own DNA, RNA and tRNA, their nucleotide synthesis also requires the assistance of the folate cycle. As described in the above paragraph, SHMT and MTHFD are important enzymes involved in the folate cycle, and they can be classified as cytosolic (SHMT1 and MTHFD1) and mitochondrial (SHMT2 and MTHFD2) forms. Despite the fact that they are located in two distinct compartments, they are transporters for folate, format, serine and glycine [93].

Although the cytosolic and mitochondrial folate cycles act in a similar way, there is some difference in glycine metabolism. SHMT can convert serine to glycine to donate a 1-carbon unit to the folate cycle. Moreover, glycine is another amino acid that can offer a 1-carbon unit in mitochondria, which is facilitated by the glycine cleavage system (GCS). GCS, also known as glycine decarboxylase complex (GDC), is loosely attached to the inner mitochondrial membrane. As a result, mitochondrial folate cycle seemed to have a better efficiency than the cytosolic one [116]. A study indicated that GDC is required to support the growth of liver tumors [117] and the carcinogenesis of lung cancer [118].

## 9. NETs Improve Biogenesis, Dynamics and Mitophagy in Cancer Mitochondria

As described in the above paragraphs, the anticancer effects from neutrophil-NETs mainly depend on their ROS toxicity to trigger damage to the mitochondria and mtDNA of cancer cells, to induce their apoptotic or necrotic death [55,56,57,58]. The Warburg effect and glucose metabolic reprogramming are speculated to compensate for the decreased mitochondrial respiration, to increase antioxidant activities, and to offer a growth advantage in human cancers. Interestingly, recent studies have revealed that the mitochondria of survived cancer cells might become intact regardless of the existence of the Warburg effect or not. Cancer cells could execute aerobic glycolysis and mitochondrial respiration facultatively [119]. Similar to the wound healing process, neutrophil-NETs could be involved in either tissue damage or tissue repair [120]. Therefore, it is of great interest to know whether the NETs would be a double-edged sword to affect the biogenesis of mitochondria in human cancer. It is an important issue and merit a further evaluation.

### 9.1. Mitochondrial DNA Regulation and Mitochondrial Biogenesis

**Figure 5:**
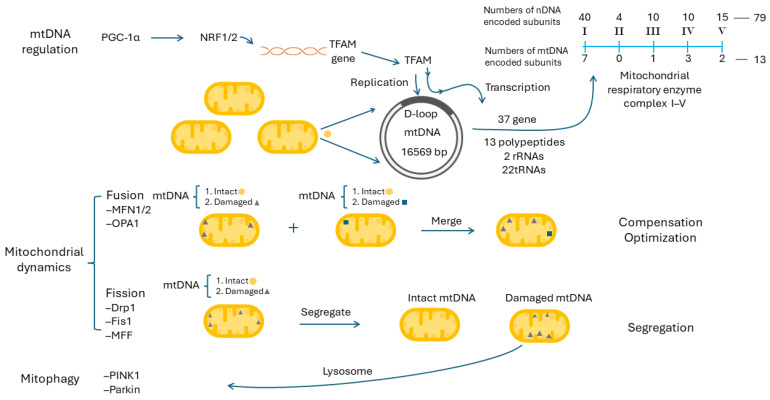
The regulation of mitochondrial biogenesis, including mtDNA regulation, mitochondrial dynamics and mitophagy, is illustrated. Drp1: dynamin-related protein 1; Fis1: human fission factor-1; MFF: mitochondrial fission factor; MFN1/2: mitofusin 1/2; mtDNA: mitochondrial DNA; nDNA: nuclear DNA; NRF1/2: nuclear respiratory factors 1/2; OPA1: optic atrophy factor 1; PGC-1α: peroxisome proliferators-activated receptor gamma coactivator-1α; MFN1/2: mitofusin 1/2; OPA1: optic atrophy factor 1; MFF: mitochondrial fission factor; PINK1: PTEN-induced kinase 1 (PINK1); TFAM: mitochondrial transcription factor A.

Human mitochondria have their own genome, mitochondrial DNA (mtDNA), and they are different from nuclear DNA (nDNA). Almost all mtDNA molecules are transmitted through the maternal lineage with a single origin; on the contrary, the nDNA are from both parents with a heterozygotic nature. There are several hundred to several thousand copies of mtDNA molecules distributed in cytoplasmic mitochondria, with 2–10 copies per mitochondrion as nucleoids to form the mitochondrial network, and they are replicated independently from the cell cycle [121]. The human mtDNA molecule is located in the IMM, consists of a non-coding displacement loop (D-loop) and a contiguous coding region, and assumes a circular double-stranded structure 16,569 base-pairs (bps) in size. Because most human mitochondrial genes have been laterally transferred to the nuclear genome during evolution, human mtDNA contains only 37 compact genes without intron, including 13 polypeptides, 2 rRNAs and 22 tRNAs [121]. Although in a small proportion, the mtDNA-encoded 13 polypeptides constitute essential parts of the mitochondrial respiratory enzyme complexes I, III, IV and V, including 7 (ND1, ND2, ND3, ND4, ND4L, ND5, and ND6; ND abbreviated for NADH dehydrogenase) in complex I, 1 (cytochrome b) in complex III, 3 (COX I, COX II and COX III, COX abbreviated for cytochrome c oxidase) in complex IV, and 2 (ATPase 6 and ATPase 8) in complex V. Except for the 13 polypeptides, all the other polypeptides constituting mitochondrial respiratory enzyme complexes I, II, III, IV and V, as well as other mitochondrial proteins, are all encoded in nDNA (Figure 5, right upper part). Encoded by two genomes, the mitochondrial respiratory enzyme complexes imply the importance of mtDNA regulation in mitochondrial biogenesis [121]. Theoretically, the capacity for mitochondrial respiration and the generation of ATP in most human cells are highly related to the quantity (copy number) of mtDNA, including immunocytes [122,123].

To maintain sufficient mtDNA copies and their expressions, several proteins, including mitochondrial RNA polymerase, mitochondrial single-stranded-DNA-binding protein, mitochondrial DNA polymerase gamma, mitochondrial DNA-directed RNA polymerase, mitochondrial transcription factor A (TFAM) and mitochondrial transcription factor B1/2, are required for the replication and transcription of mtDNA [122,124]. Among them, TFAM is unique, because it regulates both the replication and transcription of mtDNA after binding to the D-loop [125]. The expression of TFAM is regulated by nuclear respiratory factor 1 and nuclear respiratory factor 2 (NRF1 and NRF2) and peroxisome proliferators-activated receptor gamma coactivator-1α (PGC-1α) [122]. Previous studies have demonstrated that the TFAM gene contains consensus-binding sites for NRF1 and NRF2 to stimulate the synthesis of TFAM [126]. The expression levels of NRF1/2 are further regulated by activated PGC-1α, which acts as a master control in mitochondrial biogenesis [127]. The PGC-1α/NRF1/2/TFAM pathway is important in mitochondrial biogenesis. The roles of mtDNA replication and transcription in the regulation of mitochondrial biogenesis are depicted in the upper panel of Figure 5.

The roles of PGC-1α/NRF1/2/TFAM in human cancers are still under investigation. [128]. Different levels of TFAM expression have been reported in cancers. As shown in The Human Protein Atlas (available online: https://www.proteinatlas.org/, accessed on 20 August 2024), a high TFAM expression was identified as a favorable prognostic variable in patients with renal cancer and ovarian cancer, but an unfavorable one in patients with endometrial cancer. TFAM was speculated to be a tumor suppressor in renal cell carcinoma, lung cancer, gastric cancer and head and neck cancer cell lines [129,130,131,132], but a tumor promoter in esophageal cancer and colorectal cancer cell lines [133,134]. Likewise, different results of NRF expression were noted in several human cancer tissues. NRF1 can inhibit the proliferation and migration of PC3 prostate cancer cells [135], but it can drive hepatocellular carcinoma progression [136]. Concerning the upstream regulator PGC-1α, its dual roles were also reported, including as a tumor promotor in prostate cancer, colorectal cancer and breast cancer, but with a tumor suppressor role in ovarian cancer, liver cancer and melanoma [128].

### 9.2. Mitochondrial Dynamics, Mitophagy and Mitochondrial Biogenesis

Due to the absence of intron and a lack of histone protection, located in the IMM with a high concentration of ROS from electron leaks during ETC and insufficient DNA repair machinery, mtDNA molecules are more susceptible to oxidative damage and accumulate much more damage and mutations than do nDNA molecules [137]. This accounts for the necessity of thousands copies of mtDNA molecules and the coexistence of wild-type and damaged/mutant mtDNA molecules in a cell. Thus, how to keep an optimal number, the integrity and a sufficient expression of these heterogeneous mtDNA molecules, i.e., the heteroplasmic patterns, is very important. The quality and quantity of mtDNA are regulated by mitochondrial dynamics, including mitochondrial fusion and fission, and mitophagy [121,138]. Studies have revealed that mitochondrial dynamics is also related to cellular metabolic patterns, including a favoring of mitochondrial fusion to OXPHOS and mitochondrial fission to aerobic glycolysis, which contributes to mitochondrial plasticity in glucose metabolism [138].

### 9.3. Mitochondrial Dynamics and Mitophagy

Mitochondrial fusion (Figure 5, middle panel) is a process where mitochondria are merged together into a connected network. It integrates two different types of mitochondria, intact/intact mitochondria, damaged/intact mitochondria or damaged/damaged mitochondria, into a fused mitochondrion. Mitochondrial fusion can join two mitochondrial genomes with different defects/backgrounds to work together, to share their genetic information and to compensate for their individual defects within the fused mitochondria. This can optimize mitochondrial function in proper [138]. Mitochondrial fusion requires the involvement of outer mitochondrial membrane proteins, mitofusin 1/2 (MFN1/2), and the inner mitochondrial membrane protein, optic atrophy factor 1 (OPA1) [139].

Mitochondrial fission (Figure 5, middle panel) is a process to divide a mitochondrion into two daughter mitochondria, either from intact mitochondrion into intact mitochondria or from damaged mitochondrion into intact mitochondrion and damaged mitochondrion. These daughter mitochondria can be separated into smaller and fragmented ones. As a result, this can segregate the damaged mitochondria or mtDNA molecules to be removed by mitophagy, and help cells to undergo division without the incorporation of damaged mitochondria [138]. The first step to initiate mitochondrial fission is to inhibit mitochondrial fusion proteins. The second step is to recruit mitochondrial fission protein, dynamin-related protein 1 (Drp1), from the cytosol to the outer mitochondrial membrane. Drp1 can mediate mitochondrial excision by interacting with other mitochondrial fission proteins, including human fission factor-1 (Fis1), mitochondrial fission factor (MFF) and mitochondrial dynamics proteins of 49 kDa and 51 kDa [139].

In brief, MFN1/2, OPA1 and Drp1 are important proteins participating in mitochondrial dynamics. They have been evaluated in several human cancers and remain vigorously studied. Low MFN2 expression was observed in colon cancer, and high MFN2 expression has been associated with a better prognosis [140]. In breast cancer, it was discovered that an upregulation of OPA1 expression could correlate with a worse prognosis and a downregulated OPA1 could attenuate its proliferation, migration and invasion [141]. Concerning Drp1, it is significantly higher in breast cancer than in adjacent normal tissues, and a high expression level of Drp1 is related to poor survival in breast cancer patients [142].

Mitophagy (Figure 5, lower panel) is a selective process to degrade the damaged mitochondria through autophagy. Specifically, these damaged mitochondria are sequestered and then degraded by lysosome. To maintain mitochondrial integrity, cells can remove these damaged mitochondria swiftly and efficiently [138]. In mammalian cells, mitophagy regulated through the PTEN-induced kinase 1 (PINK1) and Parkin pathways are best characterized [143]. They have been appraised in several human cancers. It was demonstrated that low levels of PINK1 and Parkin expression were associated with poor prognosis in patients with esophageal squamous cell carcinoma [144]. Altered PINK1 protein expression was associated with a worse outcome in colorectal cancer with liver metastasis [145]. Downregulated PINK1 expression was also identified as an independent prognostic variable in papillary renal cell carcinoma [146].

### 9.4. NETs in Cancer Progression and Cancer in NET Formation—Reciprocal Interaction between Cancer and NETs

Based on the components of the NETs, several markers, including CitH, cell-free DNA (cfDNA), MPO, NE, the MPO-DNA complex, NE-DNA complex and PAD4, have been used to reflect the degrees of NET activation [147]. Their high levels do bear clinical implications in human cancers. Patients with advanced-stage cancers had a higher level of CitH3 and poor prognosis [148]. A high CitH3 level was applied to predict the risk of mortality in cancer patients [149]. Concerning cfDNA, a higher level of cfDNA was noted in several human cancers and was associated with a poor survival of patients [150]. In a review of 1370 clinical trials, the clinical usefulness of cfDNA was demonstrated in cancer patients, including in the detection of cancer, the real-time monitoring of acquired resistance to therapy, accurate disease progression surveillance and the improvement of treatment [151]. A high serum MPO-DNA was observed in cancers with liver metastases, and it was associated with a shorter survival [152]. Among patients with breast cancer, a higher NE was associated with a poor survival [153]. Likewise, a high NE-DNA level might be a critical factor in the poor outcome of gastric cancer patients [154]. All these results have highlighted the potential role of high NETs in cancer progression.

To verify the pathogenic role of NETs in the progression of human cancers, attention has been paid to them in the past decades. In a mouse model about the progression of breast cancer, it was demonstrated that if the breast cancer cells were surrounded by a NET-like structure, they tended to cause lung metastases; on the contrary, if the mice were treated with DNase I-coated nanoparticles to digest NETs, the abilities of cancer cells to cause lung metastases were ameliorated [155]. Because PAD4 is required in NET formation, its inhibition seems a strategy to reduce NET formation to decrease cancer progression. In an animal model for liver metastasis, metastatic tumors in PAD4-knockout mice were significantly fewer and smaller [156]. Among null mice undergoing the subcutaneous inoculation of HCT116 and Huh7 cells, the tumors would grow slower and smaller if they received DNase injection [156]. Taken together, an increased formation and activation of NET formation could contribute to human cancer progression.

**Figure 6:**
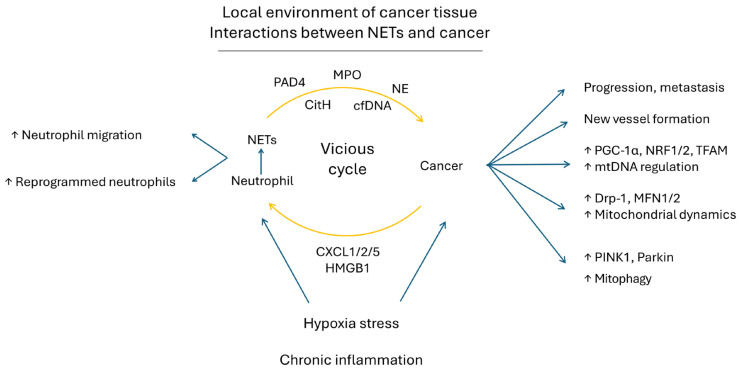
Local environment and the interactions between NETs and cancer are illustrated. CitH: citrullinated histone; cfDNA: cell free DNA; CXCL1/2/5: chemokine C-X-C motif ligand 1/2/5; Drp1: dynamin-related protein 1; HMGB1: high mobility group box 1; MFN1/2: mitofusin 1/2; MPO: myeloperoxidase; mtDNA: mitochondrial DNA; NE: neutrophil elastase; NETs: neutrophil extracellular traps; NRF1/2: nuclear respiratory factor 1/2; PAD4: protein arginine deiminase 4; PGC-1α: peroxisome proliferators-activated receptor gamma coactivator-1α; PINK1: PTEN-induced kinase 1; TFAM: mitochondrial transcription factor A.

Strategies to recruit neutrophils and induce NET formation are under investigation (Figure 6). Hypoxia stress might account for this scenario. Within the tumor environment, hypoxia is commonly present, especially in rapidly growing solid tumors, and has been linked to a chronic inflammatory state [157,158]. Secretions from human cancer cells under hypoxic stress could attract neutrophils and induce NET formation. The reciprocal interactions between NETs and tumors would sustain a vicious cycle [14,159]. In an animal model of colorectal cancer and hepatic metastases, it was found that when the liver exerted an ischemia stress, the environments would attract many more NETs to cause more liver metastases [152]. In an in vitro cell model, when neutrophils were incubated with specialized culture media, which had been obtained from cancer cell lines cultured under hypoxia conditions, the neutrophils exhibited a higher transwell migration activity and cause much more NETs formation. Interestingly, cancer cells under hypoxia could express higher mRNA levels of chemokines, including chemokine C-X-C motif ligand 1 (CXCL1)/keratinocyte-derived chemokine (KC), chemokine C-X-C motif ligand 2 (CXCL2)/macrophage inflammatory protein-2 (MIP2) and chemokine C-X-C motif ligand 2 (CXCL5)/Lipopolysaccharide-induced CXC Chemokine (LIX), which are supposed to be released to the surroundings of cancer cells to attract neutrophils [156]. Meanwhile, an increase of high mobility group box 1 (HMGB1) in the culture medium was also noted, and it is important for NET formation [156]. In brief, cancer cells can secrete chemo-attractors to recruit neutrophils and induce NET formation [155].

### 9.5. NETs Augment mtDNA Biogenesis, Mitochondrial Dynamic and Mitophagy in Cancers to Promote Their Progression

The exact roles of intra-tumor neutrophils and NETs in promoting cancer progression have been ever-increasingly recognized (Figure 6). Recently, a pioneer study demonstrated that intra-tumor neutrophils could be reprogrammed into a new type of tumor-infiltrating neutrophil and could be gathered into a single long-lived cell subset to promote the growth of new blood vessels, which could enhance cancer survival in areas with low oxygen and a limited supply of nutrients [160]. Under such a circumstance, it is of interest to know whether the cancer mitochondria may play a role in the increase of tumor growth.

Some cell line studies showed that a high mitochondrial biogenesis is closely related to the high invasiveness of cancers, as evidenced in esophageal cancer and colorectal cancer [133,134]. Moreover, an increase in mtDNA copy number was observed in the carcinogenesis of head and neck cancer and in the progression of esophageal cancer [161,162]. Despite the hypothesis of mitochondrial respiratory dysfunction by Warburg, intact mitochondria remained indispensable and important in cancer progression [163].

Interestingly, Yazdani et al. reported the positive role of NETs in the maintenance of the mitochondrial homeostasis of cancer cells and the promotion of cancer aggressiveness through cellular and animal studies [156]. They confirmed that NETs could confer a rapid tumor growth and a higher expression of PGC-1α, NRF1 and TFAM, as well as increased levels of mitochondrial density, mtDNA, ATP production and oxygen consumption rate (OCR) in cancers [156]. Besides, NETs can regulate mitochondrial dynamics and mitophagy due to higher levels of DRP-1 and MFN2, as well as PINK1 and Parkin proteins [156]. In brief, NETs can enhance the replication of mtDNA, mitochondrial biogenesis, mitochondrial dynamics and mitophagy to promote the growth of cancer cells.

## 10. Future Perspective

An important area awaiting a breakthrough is the in vitro and in vivo study of neutrophils during the progression and metastasis of cancer cells. Neutrophils are the most abundant immunocytes in the blood, and they act as the first line of defense against invading pathogens/stimuli. Nevertheless, their short life span, as well as their inability to be cryopreserved and experience clonal expansion in vitro, remain major challenges to researchers. As a result, most studies about neutrophils mainly rely on cells freshly isolated from the peripheral blood of normal subjects and patients, which can cause a variation in experimental data due to different donor characteristics. Moreover, it is more difficult to conduct research on the interaction between neutrophils and cancer cells. Therefore, how to establish a cellular model between human cancer cells and matched neutrophils warrants further investigation.

## 11. Conclusions

In this review, we have summarized and discussed the recent advances in studies on the associations between mitochondrial plasticity, glucose metabolic reprogramming and oxidative stress-elicited NET formation in human cancers. Recent advances in the multifaceted cancer metabolism in response to oxidative stress and chronic inflammation have provided us with new insights into the adaptability of cancer cells, to survive and proliferate under various conditions. How to manipulate NETs might be an opportunity for the development of novel anticancer treatments in the future.

## Data Availability

Not applicable.

## Abbreviations

3PHP, 3-phosphate hydroxypyruvate; 3PS, 3-phosphoglycerate; 3-P-serine, 3-phosphoserine; 6PG, 6-phosphogluconate; 6PGD, 6-phosphogluconate dehydrogenase; 6PGLase, 6-phosphogluconolactonase; 6PGL, 6-phosphogluconolactone; 8-OHdG, 8-hydroxy-2′-deoxyguanosine; A, adenine; ACL, ATP-citrate lyase; AD, Alzheimer’s disease; ALI, Acute lung injury; α-KG/alpha-KG, alpha-ketoglutarate; C, cytosine; cfDNA, cell-free DNA; CitH, citrullinated histone; CXCL1/2/5, chemokine C-X-C motif ligand 1/2/5; DAMPs, damage-associated molecular patterns; D-loop, displacement loop; DM, diabetes mellitus; Drp1, dynamin-related protein 1; ERK(s), extracellular signal regulated kinase(s); ETC, electron transport chain; F, folate; Fis1, human fission factor-1; FAD, oxidized flavin adenine dinucleotide; FADH_2_, reduced flavin adenine dinucleotide; FR, folate reductase; G, guanine; G6P, glucose-6-phosphate; G6PD, glucose-6-phosphate dehydrogenase; GCS, glycine cleavage system; GDC, glycine decarboxylase complex; GLS, glutaminase; GLUD, glutamate dehydrogenase; GPx, glutathione peroxidase; GR, glutathione reductase; GSH, reduced glutathione; GSSG, oxidized glutathione; HBV, hepatitis B virus; HCC, hepatocellular carcinoma; HK, hexokinase; HMGB1, high mobility group box 1; HP, *Helicobacter pylori*; HPV, human papillomavirus; IDH, isocitrate dehydrogenase; IKK, IkappaB (I-κB) kinase; IMM, inner mitochondrial membrane; IMS, intermembrane space; JNK(s), c-Jun N-terminal kinase(s); KC, keratinocyte-derived chemokine; Keap1-Nrf2-ARE, Kelch-like ECH-associated protein 1 (Keap1)/nuclear factor erythroid 2-related factor 2 (Nrf2)/antioxidant response elements (ARE); LDH, lactate dehydrogenase; LIX, Lipopolysaccharide-induced CXC Chemokine; LPS, lipopolysaccharides; MAPK(s), mitogen-activated protein kinase(s); MCT1, monocarboxylate transporter 1; MFF, mitochondrial fission factor; MFN1/2, mitofusin 1/2; MIP2, macrophage inflammatory protein-2; mtDNA, mitochondrial DNA; mtROS, mitochondrial ROS; MPO, myeloperoxidase; MTHFD, methylenetetrahydrofolate dehydrogenase/cyclohydrolase; NAD^+^, oxidized nicotinamide adenine dinucleotide; NADH, reduced nicotinamide adenine dinucleotide; NADP^+^, oxidized nicotinamide adenine dinucleotide phosphate; NADPH, reduced nicotinamide adenine dinucleotide phosphate; nDNA, nuclear DNA; NE, neutrophil elastase; NEAA, non-essential amino acids; NETs, neutrophil extracellular traps; NF-κB, nuclear factor kappa-light-chain-enhancer of activated B cells; NIK, NF-κB-inducing kinase; NOX, NADPH oxidase; NRF1/2, nuclear respiratory factor 1/2; OAA, oxaloacetate; OXPHOS, oxidative phosphorylation; OMM, outer mitochondrial membrane; OPA1, optic atrophy factor 1; PAD4, protein arginine deiminase 4; PAMPs, pathogen-associated molecular patterns; PD, Parkinson’s disease; PDH, pyruvate dehydrogenase; PEP, phosphoenolpyruvate; PGC-1α, peroxisome proliferators-activated receptor gamma coactivator-1α; PHGDH, phosphoglycerate dehydrogenase; PI3K-Akt, phosphoinositide-3-kinase (PI3K)-Akt; PINK1, PTEN-induced kinase 1; PKC, protein kinase C; PM2.5, particulate matters ≤ 2.5 µm; PMA, Phorbol 12-myristate 13-acetate; PPP, pentose phosphate pathway; PSAT1, phosphoserine aminotransferase; PSPH, 1–3-phosphoserine phosphatase; R5P, ribose 5-phosphate; RPE, ribulose 5-phosphate epimerase; RPI, ribose 5-phosphate isomerase; RPIA, RPI isomerase A; ROS, reactive oxygen species; redox, reduction and oxidation; Ru5P, ribulose 5-phosphate; SHMT, serine hydroxymethyl transferase; SOD, superoxide dismutase; SSP, serine synthesis pathway; T, thymine; THF, tetrahydrofolate; TFAM, mitochondrial transcription factor A; TS, thymidylate synthase; U, uracil; Xu5P, xylulose 5-phosphate.
